# Difficulties Experienced by Older Listeners in Utilizing Voice Cues for Speaker Discrimination

**DOI:** 10.3389/fpsyg.2022.797422

**Published:** 2022-03-03

**Authors:** Yael Zaltz, Liat Kishon-Rabin

**Affiliations:** Department of Communication Disorders, The Stanley Steyer School of Health Professions, Sackler Faculty of Medicine, Tel Aviv University, Tel Aviv, Israel

**Keywords:** speaker discrimination, older adults, fundamental frequency, formant frequencies, voice discrimination

## Abstract

Human listeners are assumed to apply different strategies to improve speech recognition in background noise. Young listeners with normal hearing (NH), e.g., have been shown to follow the voice of a particular speaker based on the fundamental (F0) and formant frequencies, which are both influenced by the gender, age, and size of the speaker. However, the auditory and cognitive processes that underlie the extraction and discrimination of these voice cues across speakers may be subject to age-related decline. The present study aimed to examine the utilization of F0 and formant cues for voice discrimination (VD) in older adults with hearing expected for their age. Difference limens (DLs) for VD were estimated in 15 healthy older adults (65–78 years old) and 35 young adults (18–35 years old) using only F0 cues, only formant frequency cues, and a combination of F0 + formant frequencies. A three-alternative forced-choice paradigm with an adaptive-tracking threshold-seeking procedure was used. Wechsler backward digit span test was used as a measure of auditory working memory. Trail Making Test (TMT) was used to provide cognitive information reflecting a combined effect of processing speed, mental flexibility, and executive control abilities. The results showed that (a) the mean VD thresholds of the older adults were poorer than those of the young adults for all voice cues, although larger variability was observed among the older listeners; (b) both age groups found the formant cues more beneficial for VD, compared to the F0 cues, and the combined (F0 + formant) cues resulted in better thresholds, compared to each cue separately; (c) significant associations were found for the older adults in the combined F0 + formant condition between VD and TMT scores, and between VD and hearing sensitivity, supporting the notion that a decline with age in both top-down and bottom-up mechanisms may hamper the ability of older adults to discriminate between voices. The present findings suggest that older listeners may have difficulty following the voice of a specific speaker and thus implementing doing so as a strategy for listening amid noise. This may contribute to understanding their reported difficulty listening in adverse conditions.

## Introduction

Older people often find it extremely difficult to understand speech and converse in noisy environments, particularly when the noise includes several speakers (e.g., Pichora-Fuller, 1997). Such difficulties may limit their ability to participate in social, occupational, and educational activities, isolating them from their families and friends (Cacciatore et al., 1999; Arlinger, 2003; Pronk et al., 2011; Gopinath et al., 2012). Many studies have attempted to assess the contribution of different factors to the difficulties older adults experience in speech perception amid noise and found that they explain the results only in part. These include elevated hearing thresholds (e.g., Jayakody et al., 2018), reduced frequency and temporal resolution (e.g., Anderson and Karawani, 2020), and declining cognitive functioning (e.g., Pichora-Fuller and Singh, 2006; Schneider et al., 2010). One approach to further our understanding of the difficulties experienced by older adults in noisy conditions is to assess their ability to apply listening strategies that are known to assist younger adults in listening amid noisy backgrounds. One such strategy includes identifying and following the acoustic voice cues of a target speaker, such as the fundamental frequency (F0) and formant frequencies of their voice (e.g., Bronkhorst, 2015). Young adults have been shown to efficiently implement this strategy to segregate the relevant and irrelevant speakers (e.g., Bronkhorst, 2015), relying on efficient spectral (formants) and temporal (F0) processing of the speech signal (e.g., Fant, 1960; Lieberman and Blumstein, 1988; Carlyon and Shackleton, 1994; Fu et al., 2004; Oxenham, 2008; Xu and Pfingst, 2008). Given that spectro-temporal processes are known to degrade with age (e.g., Vongpaisal and Pichora-Fuller, 2007; Souza et al., 2011; Schvartz-Leyzac and Chatterjee, 2015; Chintanpalli et al., 2016; Goupell et al., 2017; Anderson et al., 2021) it may be difficult for older adults to take advantage of differences in F0 and/or formant information for talker segregation. Age-related cognitive decline in executive functions, including attention, inhibition, and working memory (e.g., Mitchell et al., 2000; Salthouse, 2000; Harada et al., 2013), may add to the difficulty in utilizing these acoustic cues for voice discrimination. Thus, the goal of the present study was to examine the use of F0 and formant cues for voice discrimination and to assess the contribution of sensory and cognitive factors to this discrimination in older adults with normal hearing for their age (NHA), compared to young adults.

Multi-talker situations are particularly challenging for speech understanding because they force the listener to cope with both energetic (Brungart, 2001; Ezzatian et al., 2012) and informational masking (Durlach et al., 2003; Hoen et al., 2007; Ezzatian et al., 2012). Energetic masking occurs when the energy of the frequencies of the competing voices overlap those of the target voice, activating similar areas along the basilar membrane. Informational masking occurs because the competing speech may invoke related linguistic activity and/or divert attention from the target speech, interfering with the processing of the speech signal at higher linguistic or cognitive levels and making it difficult for the listener to focus on the auditory stream of interest and ignore non-relevant sounds (also known as the “cocktail party” effect, e.g., Kattner and Ellermeier, 2020). Therefore, to function well in multi-talker situations, the listener has to efficiently utilize both bottom-up (e.g., spectral separation) and top-down (e.g., focused attention) mechanisms. However, spectral and temporal processing have been shown to degrade with age, even in listeners with audiograms within normal hearing (Moore and Peters, 1992; Vongpaisal and Pichora-Fuller, 2007; Schvartz-Leyzac and Chatterjee, 2015; Chintanpalli et al., 2016; Goupell et al., 2017). A negative age effect has also been reported for higher cognitive abilities, such as executive functions, including memory, attention, and inhibition (e.g., Mitchell et al., 2000; Salthouse, 2000; Harada et al., 2013). The poor speech-in-noise understanding of older NH adults may, therefore, be the result of varying degrees of decline in their peripheral, linguistic, and/or central and cognitive processing (e.g., Working Group on Speech Understanding and Aging, 1988; Humes et al., 2012; Tremblay et al., 2021).

One listening strategy that is assumed to assist in segregating the target voice from competing non-relevant sounds is to identify and track the acoustic characteristics of the voice of the speaker of interest (e.g., Bronkhorst, 2015). These characteristics include fundamental frequency (F0), which is influenced by the length, mass, and rate of vibration of the vocal cords, and formant frequencies (i.e., resonant frequencies of the vocal tract), which are influenced by the vocal tract length (VTL; e.g., Darwin et al., 2003; Vestergaard et al., 2009, 2011; Mackersie et al., 2011; Schvartz-Leyzac and Chatterjee, 2015; Başkent and Gaudrain, 2016). Both of these cues provide robust information regarding the speaker, such as age and gender, as well as idiosyncratic characteristics that are unique to that speaker and his/her personality, nearly as unique as our fingerprints according to some researchers (Shultz, 2015). Studies in young adults and children have shown that these listeners rely heavily on both types of cues (F0 and formant frequencies) to discriminate among (Zaltz et al., 2020) and segregate talkers (e.g., Darwin et al., 2003) as well as to identify the gender of a specific speaker (e.g., Smith and Patterson, 2005; Smith et al., 2007; Skuk and Schweinberger, 2014; Başkent and Gaudrain, 2016). Moreover, listeners who have difficulty perceiving differences in F0 and formant frequencies, such as cochlear implant users, showed reduced voice discrimination, which may explain, at least in part, their poor performance when listening amid noise (e.g., Gaudrain and Başkent, 2018; Zaltz et al., 2018).

Studies suggest that F0 coding relies primarily on efficient processing of the temporal envelope and/or of the temporal fine-structure cues of the signal, whereas formant coding primarily involves place coding of spectral energy peaks (e.g., Fant, 1960; Lieberman and Blumstein, 1988; Carlyon and Shackleton, 1994; Fu et al., 2004; Chatterjee and Peng, 2008; Oxenham, 2008; Xu and Pfingst, 2008). Previous studies have shown the effect of age on F0 discrimination (e.g., Moore and Peters, 1992; Vongpaisal and Pichora-Fuller, 2007; Souza et al., 2011; Anderson et al., 2021). It has been shown, for example, that older adults require twice the difference between F0s to reach similar accuracy in concurrent vowel identification with harmonic complexes and synthetic vowels, compared to their younger peers (Moore and Peters, 1992; Vongpaisal and Pichora-Fuller, 2007). Electrophysiological studies have demonstrated pronounced reductions in phase locking in older adults, suggesting reduced neural synchrony among this population (e.g., Anderson et al., 2021). These findings were interpreted to reflect impaired periodicity coding in older listeners (Schvartz-Leyzac and Chatterjee, 2015), which, in turn, may negatively influence the utilization of F0 cues for talker discrimination in this population. Other studies have showed the effect of age on the utilization of formant changes for vowel identification (Vongpaisal and Pichora-Fuller, 2007; Chintanpalli et al., 2016; Goupell et al., 2017). However, no study, to our knowledge, has specifically investigated the ability to use changes in F0, formants and their combination for speaker discrimination.

Efficient utilization of the relevant acoustic cues for talker discrimination may also require complex cognitive processing, such as, attending to F0 and formant information of the different talkers, and storing this information in memory for decision making and for future reference. Therefore, in older adults, the reported age-related deterioration in the ability to focus attention on the relevant features of the stimulus while inhibiting the processing of non-relevant features (McDowd and Shaw, 2000; Schneider et al., 2007; Harada et al., 2013), may negatively affect their ability to discriminate between speakers based on specific voice cues. Similarly, decline in working memory processes with age, including poor short-term maintenance and manipulation of information during encoding (e.g., Mitchell et al., 2000), and/or general slowing of cognitive processes (Salthouse, 2000), may add to difficulties in utilizing F0 and formant cues for talker identification and stream segregation. Support for this hypothesis can be found in a recent study where the authors argued that poor talker identification amid noise in older adults may have been related to their difficulty to learn and store in memory the voice information associated with a particular speaker (Best et al., 2018). It is possible that a simpler task that examines the perception of F0 and formant frequencies *via* discrimination rather than *via* identification may better assess the utilization of voice cues in older adults. Thus, the present study aimed to assess the use of F0 cues alone, formant cues alone, and the combination of F0 and formants in a VD task in older adults with NHA, and to compare their performance to that of young adults with NH. In addition, because even a simple discrimination task may require attention and working memory capabilities, our second aim was to assess the contribution of higher cognitive abilities, specifically, executive control abilities and working memory, to VD performance.

## Materials and Methods

### Participants

**Figure 1:**
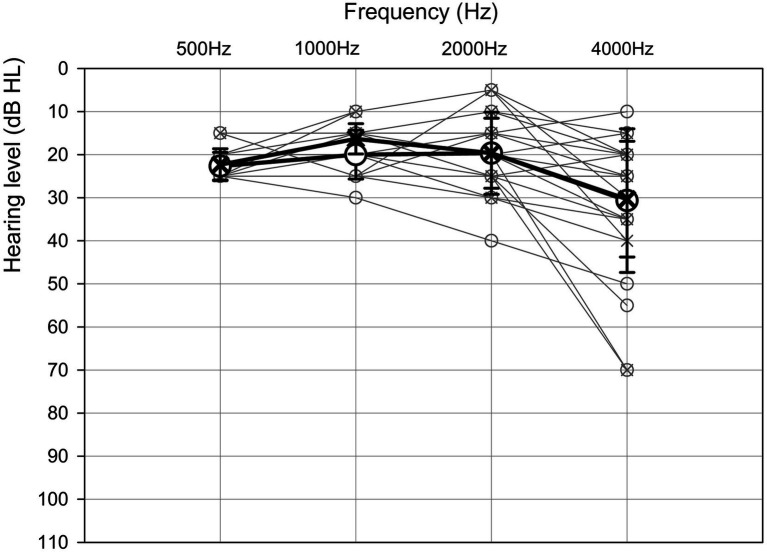
Mean (thick black lines and symbols; ± 1 standard deviation) and individual (thin gray lines and symbols) hearing thresholds at 500 Hz, 1,000 Hz, 2000 Hz, and 4,000 Hz for the right (circle) and left (cross) ears for the older adults (*n* = 15).

A total of 50 participants were recruited for the present study: 15 older adults (65–78 years, mean = 68.93 ± 3.63 years; median = 68) and 35 young adults (18–35 years, mean = 22.29 ± 3.16 years; median = 22). The VD results of 15 participants from the young-adult group were previously reported (Zaltz et al., 2020). For the current study, we tested an additional 20 young adults to obtain a larger dataset for comparison. As no significant difference in age or test results was observed between the two groups of young adults (*p* > 0.05), their data were combined for all further analyses. The young adults had hearing sensitivity within the normal range in both ears, with pure-tone air conduction thresholds <20 dB HL at octave frequencies of 500–4,000 Hz (Ansi, 2018). For the older adults, eight participants had thresholds less or equal to ≤25 dB HL, five participants had thresholds less or equal to ≤40 dB HL, one had thresholds less or equal to ≤55 dB HL, and one had thresholds less or equal to ≤70 dB HL at octave frequencies of 500–4,000 Hz (Figure 1). Overall, pure-tone air conduction thresholds for the older adults were within the normal range for their age (Engdahl et al., 2005), with a pure-tone average across ears and four frequencies (PTA4) of less than 33 dB HL. None of the participants had any previous psychoacoustic experience in similar tasks, they had no known history of ear disease, and they had completed at least 12 years of formal education. All participants were fluent in Hebrew. Six of the older participants were native Hebrew speakers. The other nine older adults immigrated to Israel at a mean age of 24 (±11) years (range: 3–43 years), and thus were exposed to Hebrew for an average of 45 (±11) years (range of exposure: 29–72 years). Their mother tongues were French (*n* = 4), Arabic (*n* = 3), English (*n* = 1), and Romanian (*n* = 1). All older adults had cognitive ability levels within the normal range (Mini Mental State Examination score ≥ 27; based on the English version; Folstein et al., 1975), lived independently, and led an active life based on self-report. Informed consent was obtained from all participants. The study was approved by the Institutional Review Board of Ethics at Tel Aviv University.

### Stimuli

The VD test included three shortened (3-word) sentences from the Hebrew version of the Matrix sentence test. All the sentences had a simple grammatical structure (noun, verb, adjective) from a vocabulary that is appropriate for 5 year olds, and were recorded by a native Hebrew female speaker (Bugannim et al., 2019), similar to Zaltz et al. (2020). Sentences were manipulated using a 13-point stimulus continuum, exponentially ranging in √2 steps from a change of −0.18 semitone to a change of −8 semitones. This manipulation was conducted in three separate dimensions: (1) F0, (2) formant frequencies, and (3) combined F0 + formants (Zaltz et al., 2020). For a detailed explanation of the stimuli for the VD test, see Appendix A.

#### Voice Discrimination Test

A three-interval, three-alternative forced-choice procedure was used to estimate VD based on difference limen (DL) for F0 cues, formant cues, and combined F0 + formant cues. A two-down one-up adaptive tracking procedure yielded DLs corresponding to a 70.7% detection threshold on the psychometric function (Levitt, 1971). Each trial consisted of two reference sentences and a comparison sentence, specified at a random interval. Inter-stimulus interval was 300 milliseconds. When a sentence was presented, one of the three squares on the PC monitor was highlighted to correspond to that sentence. The participant was instructed to select the sentence that “sounded different” by using the mouse to click on the corresponding square or telling the tester which sentence (1, 2, or 3) “sounded different.” The same sentence was used for each VD threshold, resulting in a change only in the tested acoustic cue (F0, formants, or F0 + formants). No feedback was provided, and there was no time limit for responding. The difference between the stimuli was reduced by a factor of two until the first reversal and then reduced or increased by √2 until the sixth reversal. DLs were calculated as the arithmetic mean of the last four reversals.

### Cognitive Tests

Cognitive capability was assessed only for older adults using the Hebrew version of the Mini Mental state examination test (based on the English version; Folstein et al., 1975). This test examined several mental functions, including orientation, memory, attention, naming, understanding of oral and written instructions, drawing, and writing. Overall, the test included 11 questions and lasted approximately 10 min. A score of 27 points or higher indicated cognitive ability within the normal range (Folstein et al., 1975).

Auditory working memory was assessed for all participants using a recorded version of the backward digit span subtest of the Wechsler Intelligence Scale (Wechsler, 1991). In the digit span test, the participants heard sequences of numbers (e.g., 2, 6, 4, and 3) and were asked to repeat them verbally in the reverse order. The passing criterion to proceed to the next longer sequence was one successful repetition of a sequence of a specific length. The score represented the number of correctly repeated sequences.

A combined effect of executive control abilities, mental flexibility, and the perceptual speed of processing was assessed for all participants using the Trail Making test (TMT) part A and part B (e.g., Bowie and Harvey, 2006; Sánchez-Cubillo et al., 2009). In part A of the test, the participants were instructed to manually connect consecutive numbers from 1–24 by drawing a line as quickly and accurately as possible. In part B of the test, the participants were instructed to manually connect a set of 24 consecutive numbers and letters (e.g., 1A, 2B, 3C…) in sequential order as quickly as possible while maintaining accuracy. If a participant made an error, the tester corrected the response before moving on to the next dot. The scores for the TMT parts A and B represent the time taken for the participant to complete the test accurately (in seconds).

### Study Design

All participants took part in a single testing session. At the beginning of the session, each participant from the older adult group was tested using the Mini Mental test. All participants performed three VD thresholds, one with each voice cue (F0, formants, F0 + formants), with a different sentence presented for each cue. Voice cues and sentences were randomized across participants. Before formal testing, each participant performed a short familiarization task with each of the voice cues to ensure that the task was understood. After the completion of VD testing, cognitive tests were conducted. Overall, the testing lasted approximately 45–50 min, including two short breaks of 5–8 min. The participants were not compensated for their time.

### Apparatus

The stimuli were delivered using a laptop personal computer through an external sound card and a GSI-61 audiometer to both ears *via* THD-50 headphones at approximately 35–40 dB SL above individual PTA4. The testing session took place in a sound-treated single-walled room.

### Data Analysis

All of the VD data were log-transformed for ANOVA to normalize the distribution of the residuals (Kolmogorov–Smirnov test: *p* > 0.05) and allow for parametric statistics. *Post hoc* analyses were conducted using Bonferroni corrections for multiple comparisons. Pearson’s coefficient correlations were conducted on the raw data. Corrections for multiple testing (ANOVA and correlations) were applied using the False Discovery Rate method. Statistical analyses were conducted using the SPSS-20 software.

## Results

**Figure 2:**
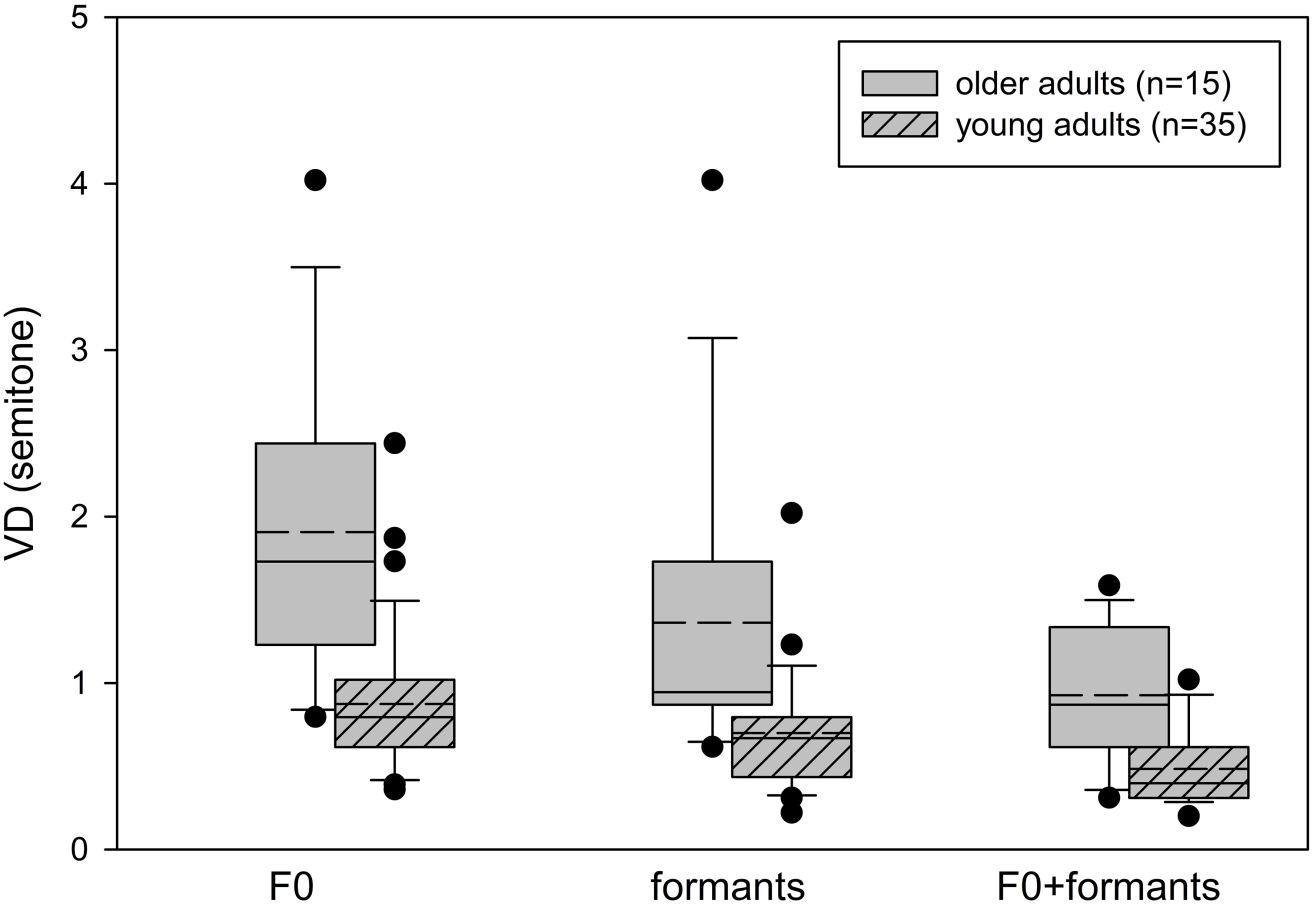
Box plots for voice discrimination thresholds with the F0, formant, and combined F0 + formant cues for the young adults (*n* = 35) and the older adults (*n* = 15). Box limits include the 25th to 75th percentile data, the solid line within the box represents the median, and the dashed line represents the mean. Bars extend to the 10th and 90th percentiles. Black dots represent outliers.

Box whisker plots of the VD thresholds based on F0, formant, and F0 + formant cues for the young and older adult participants are shown in Figure 2. The older adults’ thresholds were higher (i.e., worse) than those of the young adults for all voice cues. Two-way repeated measures ANOVA with Age as a between-subject variable and Cue (F0, formants, F0 + formants) as a within-subject variable revealed a significant effect of Age [*F*(1,48) = 27.060, *p* < 0.001, *ƞ*^2^ = 0.361], with the young adults showing better VD thresholds (*M* = 0.69 ± 0.38), compared to the older adults (*M* = 1.40 ± 0.85). There was a significant effect of Cue [*F*(2,48) = 46.056, *p* < 0.001, *ƞ*^2^ = 0.490] with no significant Age*Cue interaction [*F*(2,48) = 0.439, *p* = 0.646, *ƞ*^2^ = 0.009]. A pairwise comparison revealed better thresholds across groups with the formant cues (*M* = 0.90 ± 0.63), compared to the F0 cues (*M* = 1.18 ± 0.78; *p* = 0.011), with the best thresholds achieved with the combined F0 + formant cues (*M* = 0.62 ± 0.35; F0 > F0 + formants, *p* < 0.001; Formants > F0 + formants, *p* = 0.001). Contrast analysis showed that both groups benefited similarly from the combined cues, compared to a single cue, with no significant Age*Cue interactions for F0, compared to F0 + formants [*F*(1,48) = 0.656, *p* = 0.422], or for formants, compared to F0 + formants [*F*(1,48) = 0.01, *p* = 0.978].

**Figure 3:**
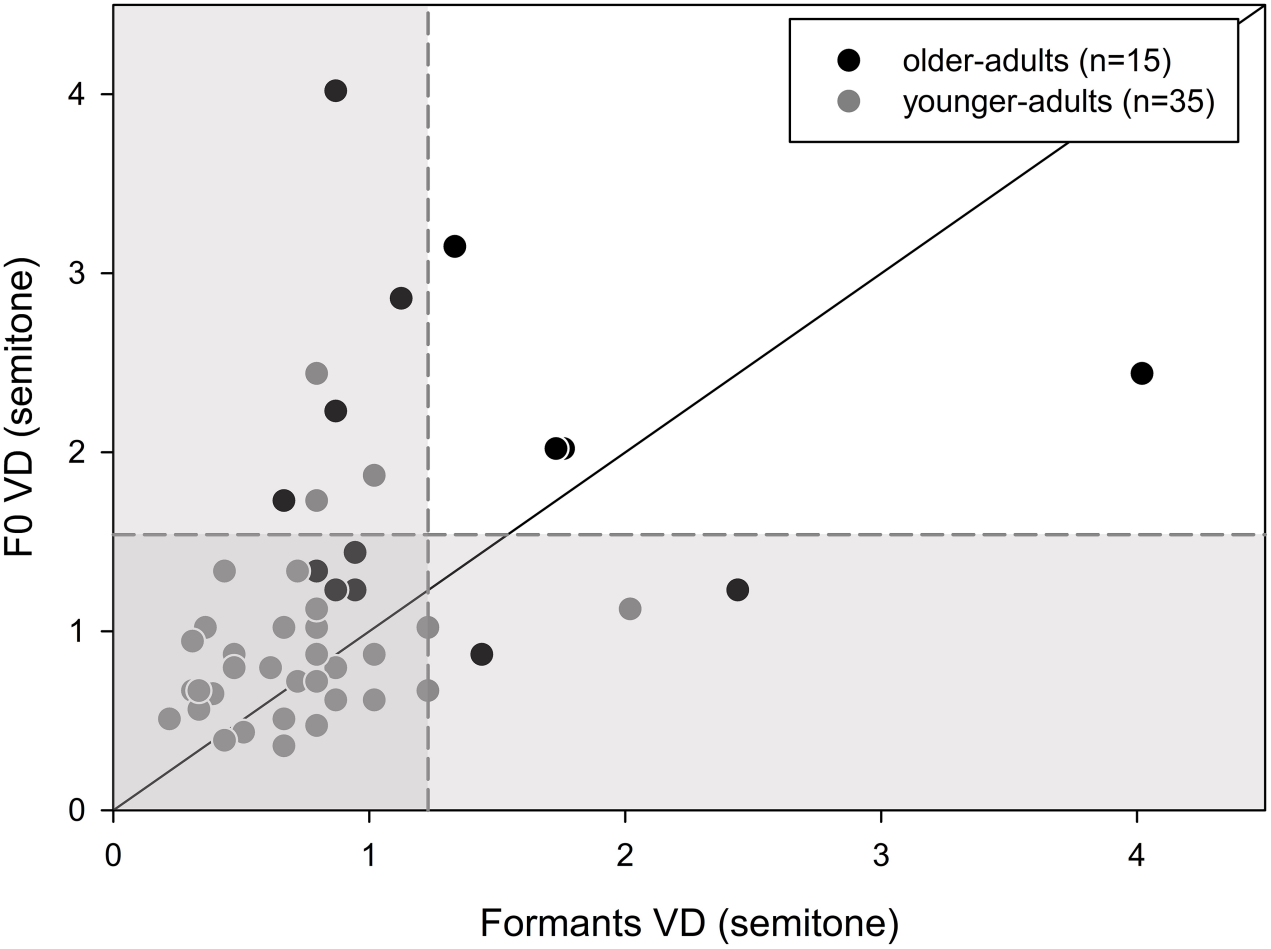
Individual discrimination thresholds (DLs) for formants vs. F0 for the young (*n* = 35) and older adults (*n* = 15) The diagonal line is positioned at x = y. Data above the line show better thresholds for the formant cues, compared to the F0 cues, whereas data below the line show better thresholds for the F0 cues. The gray area represents the mean (±1.5 standard deviation) of the young adults’ performance.

Mauchly’s test of sphericity revealed larger between-subject variability in the VD results for older adults than for young adults (*p* = 0.019). Thus, further analysis of the results was conducted at the individual level. Individual VD thresholds for F0 in relation to the formant cues for both young and older adults are shown in Figure 3. Also shown is the mean VD ± 1.5 standard deviation of the young adults (gray areas). The majority of the participants demonstrated better discrimination for formant cues, compared to F0 cues, with a higher proportion of this preference in older adults (*n* = 12, 80%) compared to younger adults (*n* = 20, 57%). However, this difference was not significant (Fisher’s exact test: *p* = 0.199). Furthermore, although group analysis showed significantly worse thresholds for the older adults, individual analysis revealed that nine (60%), seven (47%), and five (33%) of the 15 older adults performed within the range of the young adults for the formant, F0, and formant+F0 conditions, respectively.

**Figure 4:**
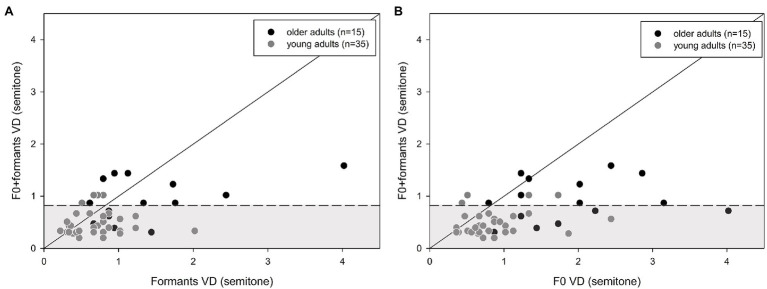
Individual discrimination thresholds (DLs) for young (*n* = 35) and older adults (*n* = 15): **(A)** shows VD for F0 vs. F0 + formants, and **(B)** shows VD for formants vs. F0 + formants. The diagonal line is positioned at x = y. Points that are located on the line show that combining the cues did not change (improve or worsen) discrimination thresholds compared to only one cue. Data above the line show the advantage of the combined cues, compared to only one acoustic cue. Data below the line demonstrate a degrading effect of the combined cues, compared to one cue. The gray area represents the mean (±1.5 standard deviation) of the young adults’ performance with the combined cues.

A comparison between the combined F0 + formant and single F0 or formant cues (Figures 4A,B) revealed that the majority of the participants benefited from the integration of two cues, compared to a single cue for VD, as is shown in Figure 4, by more data points below the diagonal line than above it. Specifically, for the older adults, 13 (87%) and 11 (73%) participants benefited from the combined cues over F0 only or formants only, respectively. For the young adults, 31 (89%) and 24 (69%) participants benefited from the combined cues over F0 only and formants only, respectively. There was no significant difference in proportions between older and younger participants (Fisher’s exact test: *p* > 0.05). However, only six (40%) older adults performed within the range of the young adults with combined cues (Figures 4A,B).

### Cognitive Abilities, Hearing Sensitivity, and Voice Discrimination

**Figure 5:**
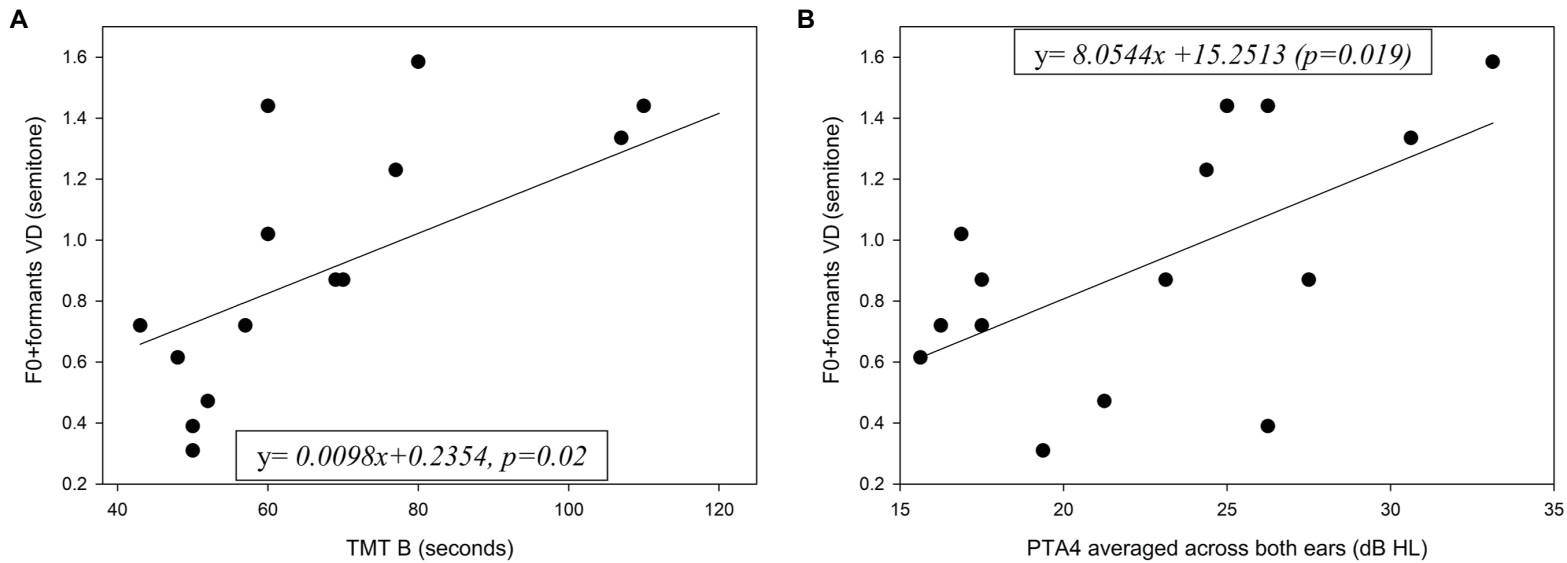
Individual F0 + formants VD for the older adults as a function of **(A)** performance on the TMT B test and **(B)** hearing sensitivity. PTA4 = mean pure-tone thresholds at 500–4000 Hz, averaged across both ears. Also shown in solid line for each graph is the function best fitting the data.

All the older adults exhibited cognitive performance in the normal range on each cognitive measure. The mean results of the cognitive tests for the young and older adults are shown in Table 1, along with the results of a one-way ANOVA comparing the two groups. Significantly better scores were achieved by the younger group in all cognitive tests. Pearson coefficient correlations were conducted separately for each group to test associations between the cognitive scores and age with the VD performance. For the older adults an additional Pearson coefficient correlation was conducted between hearing sensitivity (PTA4 averaged across both ears) and the VD performance. The full correlation results for these tests are shown in Table 2. For older adults, significant correlations were found in the combined F0 + formant testing condition between VD thresholds and TMT B scores [*r*(13) = 0.592, *r*^2^ = 0.35, *p* = 0.02] (Figure 5A), and between VD thresholds and hearing sensitivity [*r*(13) = 0.594, *r*^2^ = 0.35, *p* = 0.02] (Figure 5B). That is, shorter (i.e., better) TMT B times and better hearing sensitivity were associated with lower (i.e., better) VD thresholds when using F0 + formant cues. No significant association was found between TMT B scores and PTA4 (*p* > 0.05) for this group. No significant associations were found between cognitive abilities and VD results for the young adults (*p* > 0.05). The magnitudes of associations between F0 + formant VD and TMT B in the two groups (TMT_B X group interaction) was found statistically significant (*p* = 0.026), adding *R*^2^ = 0.063 to the proportion of explained variance in F0 + formant VD.

**Table 1:** Mean performance for each of the cognitive tests for the young adults (*n* = 35) and older adults (*n* = 15).

	Young adults	Older adults	One-way ANOVA
Wechsler digit-span (#)	5.56 (±2.02)	4.4 (±1.12)	*F*(1,48) = 4.32 *p* = 0.043
TMT A (seconds)	22.46 (±7.87)	38.8 (±15.38)	*F*(1,49) = 23.92 *p* < 0.01
TMT B (seconds)	44.16 (12.10)	70.2 (±24.38)	*F*(1,48) = 25.22 *p* < 0.01

In parentheses are ± one standard deviation. Also shown are the results of the statistical analysis comparing the two groups of participants.

**Table 2:** Pearson coefficient correlations between the cognitive scores, age, and hearing sensitivity (PTA4 averaged across both ears, available only for the older adults) and VD performance, separately for the young adults (*n* = 35), and older adults (*n* = 15).

	Young adults			Older adults		
	F0	Formants	F0 + formants	F0	Formants	F0 + formants
TMTA	−0.176 (0.311)	0.235 (0.174)	−0.137 (0.432)	0.197 (0.481)	−0.016 (0.955)	0.327 (0.235)
TMTB	−0.315 (0.070)	−0.043 (0.811)	−0.069 (0.700)	−0.092 (0.745)	0.170 (0.546)	0.592^*^ (0.020)
Wechsler digit-span	0.193 (0.274)	0.240 0.172	0.224 (0.202)	0.095 (0.736)	0.129 (0.647)	0.054 (0.848)
Age	0.060 (0.731)	0.165 (0.345)	−0.085 (0.626)	−0.359 (0.189)	−0.017 (0.953)	0.081 (0.775)
PTA4	–	–	–	0.149 (0.596)	0.413 (0.126)	0.594^*^ (0.020)

Significance level is shown in parentheses.

* Correlation is significant at the 0.05 level.

TMT, Trail Making Test.

## Discussion

In the present study, we examined the utilization of F0 and formant cues for VD in 15 older adults (65–78 years old) as compared to 35 young adults (18–35 years old). The results support the following findings: (a) Despite being generally high functioning, including hearing sensitivity expected for their age and normal cognitive function, the older adults as a group showed reduced ability to utilize voice cues (F0, formant frequencies, and the combined F0 + formants) for VD, compared to young adults; (b) However, individual analysis revealed large between-subject variability for the older adults, with 47–60% of them reaching VD performance that was within the range of young adults (mean ± 1.5 SD) with at least one acoustic cue; (c) An advantage was observed for formant cues, compared to F0 cues, for both young and older adults, in keeping with previous findings for children and young adults (Zaltz et al., 2020) and confirming that formants remain the reliable cue for VD throughout life; (d) Both the young and older participants benefitted more from the provision of the combined F0 + formant cues than from the provision of a single cue, supporting the hypothesis that older adults are capable of integrating spectral and temporal cues; (e) For the older adults, a combined effect of executive control abilities and speed of processing (as assume to be reflected by TMT B), and hearing sensitivity (PTA4) contributed significantly to the variance of VD in the combined F0 + formant condition, emphasizing the importance of basic requirements of bottom-up and top-down capabilities to allow for more advanced processing such as integration of acoustic cues.

Our major finding that as a group, older adults achieved larger (i.e., poorer) VD thresholds using F0 and formant frequency cues compared to young adults can be partly explained by age-related decline in cognitive abilities including a combined effect of processing speed, mental flexibility and executive control abilities. Normal aging is expected to include neurocognitive changes in working memory, attention, inhibition, and the speed of processing (e.g., Mitchell et al., 2000; Salthouse, 2000; Harada et al., 2013). These may be critical for focusing attention on the relevant acoustic cues for VD and storing this information in memory long enough for decision making. The significant positive association found for the older adults between the F0 + formant VD and TMT B results may suggest that attention focusing, inhibition and perceptual speed of processing played an important role in VD.

Another explanation for the poorer VD performance of the older adults may stem from the significant association that was found between hearing sensitivity (PTA4) and DL for the F0 + formant condition in the older group. This association suggests that regardless of our attempts to compensate for loss in audibility by presenting the stimuli at 35–40 dB above the average individual hearing thresholds (PTA4), older adults with poorer audiograms showed inferior VD performance. This finding may support the notion that audibility is necessary but not sufficient for good auditory processing and that resolving capabilities in the spectral and temporal domains are needed (e.g., Schneider et al., 2010). Indeed, spectro-temporal processing has been suggested to decline with age as a result of numerous deficits, including a subclinical loss of outer hair cells, broadened auditory filters, strial dysfunction, cochlear synaptopathy, and loss of neural synchrony (for a review, see Anderson and Karawani, 2020). Although temporal and spectral processing capabilities were not directly assessed in the current study, previous data suggest that F0 and formant frequency coding relies on efficient utilization of both temporal and spectral information (e.g., Fant, 1960; Lieberman and Blumstein, 1988; Carlyon and Shackleton, 1994; Fu et al., 2004; Chatterjee and Peng, 2008; Oxenham, 2008; Xu and Pfingst, 2008). Hence, deficits in the spectro-temporal processing, such as impairments in periodicity and fine-structure perception (Souza et al., 2011) may explain the poor performance in VD of older adults with greater loss of hearing sensitivity despite attempts to compensate for this loss of audibility.

One can argue that our finding of poorer VD for the older participants may have also been related to the fact that the VD test was conducted in Hebrew, which was the mother tongue for six of the 15 older participants, but for all the young participants. This hypothesis is based on the notion that testing in a second language (L2) may have increased working memory demands, and in our case, influencing VD performance for those older participants. We believe, however, that this explanation is less likely, because the stimuli for the VD task in the current study included only three sentences, one for each VD assessment. Moreover, for each threshold estimation, the same sentence was used. The listeners were, therefore, required to identify the odd sentence based only on psychoacoustic perception, with no linguistic decisions to be made. Furthermore, all three sentences were taken from the Hebrew version of the Matrix sentence test which comprises words that are suitable for 5-year-old Hebrew speakers (Bugannim et al., 2019), and included three words with a simple grammatical structure (noun, verb, adjective). Given that the nine older adults in our study whose mother tongue was not Hebrew were all fluent Hebrew speakers who were exposed to Hebrew for at least 29 years, with an average of 45 years, linguistic knowledge was probably not a contributing factor to the working memory demands of the VD task.

A second outcome of the present study is that the majority of older adults showed better discrimination thresholds with formant cues than F0 cues. This is similar to what was found in our young adults and in line with a recent study of school-age children (Zaltz et al., 2020). The advantage of formant cues for VD (in comparison to F0 cues) may be related to the amount of variation that each acoustic cue has in natural speech. While F0 varies significantly in terms of time for conveying prosodic information (standard deviation is approximately 3.7 semitones), formant frequencies are relatively constant over the duration of the vowel (standard deviation of approximately one semitone; Kania et al., 2006; Chuenwattanapranithi et al., 2008). The finding that formant frequencies remain a reliable cue for VD across the lifespan may also suggest faster degradation of temporal (F0) processing compared to spectral (formants) processing with age (Vongpaisal and Pichora-Fuller, 2007; Chintanpalli et al., 2016; Goupell et al., 2017).

Our finding that older adults benefit from combined cues to a similar degree as young adults supports the notion that older adults are able to integrate information from separately coded processes and use it to their advantage. Thus, despite the fact that formant frequencies were more easily accessed by older listeners, compared to F0 cues, this latter information was not ignored and was used to support the dominant channel of information for VD as long as their combined executive control and perceptual processing abilities were efficient (supported by the significant association found with TMT B; Bowie and Harvey, 2006; Sánchez-Cubillo et al., 2009).

Finally, our study showed a large between-subject variability in VD performance in the older group, with approximately half of the older adults showing a performance comparable to that of the young adults with at least one acoustic cue and 40% in the combined-cue condition. This variability was likely related to the considerable inter-individual variability reported for older adults in a range of auditory processing abilities and cognitive processes (e.g., Tun et al., 2012), as reflected by the significant associations found between VD performance and cognitive (TMT B) and sensory (PTA4) abilities. Given that the efficient discrimination and integration of F0 and formants are essential for speech segregation (e.g., Darwin et al., 2003; Vestergaard et al., 2009), age-related declines in cognitive and sensory processing that negatively affect these abilities may lead to impaired speech perception in noise.

## Limitations of the Study and Suggestions for Future Studies

Although we tested a relatively homogeneous group of older adults (all with normal audiograms for their age, normal mental capabilities, relatively high functioning, and similar audibility of the stimuli) and assessed several cognitive abilities in addition to VD performance, our findings provided only a partial explanation for the poor VD performance found in older adults. Psychoacoustic tests and more sensitive working memory tests may be included in future studies to further assess the relationship between temporal and spectral abilities and VD in older adults, and better reflect the correlations with age-related declines in cognitive abilities. Also, although the differences in audibility between the young and older adults were compensated by presenting the stimuli at approximately 35–40 dB SL above the individual PTA4, one cannot rule out the possibility that the inferior VD performance of the older adults was related to their poor hearing sensitivity. We believe that this issue did not have a major effect on the VD results because the acoustic cues for F0 and formants are mainly in the low-mid frequency range. However, a different or additional compensation method, such as frequency-shaped amplification on the stimuli (e.g., Souza et al., 2011) may have better accommodated the high-frequency threshold elevations of some of the older listeners. Finally, future studies may want to control the mother tongue of the participants in order to assure that there is no effect of the mono versus multilingualism status of the participants on VD performance.

## Conclusion

The present study is the first to test the contribution of F0, formant, and the combination of F0 and formant cues to VD in older adults with no hearing loss other than loss of hearing sensitivity as expected to decline with age. The findings indicate that many of the older adults found the VD task more difficult than NH young adults, presenting poorer VD thresholds across conditions. The VD thresholds in the F0 + formant condition for the older adults were associated with their audiograms, likely reflecting the importance of good bottom-up input and processing for efficient utilization of the acoustic voice cues of the talker. VD thresholds were also associated with TMT B scores, which are assumed to provide a general measure of executive control abilities, and perceptual processing, possibly reflecting their need to resort to cognitive resources in the presence of inefficient spectro-temporal processing (Schneider, 2011). These findings may explain the difficulties that older adults have in segregating talkers. The findings may also provide a possible explanation for the difficulty older adults face when listening to speech in a multi-talker environment. However, these assumptions need to be confirmed in future studies.

## Data Availability Statement

The raw data supporting the conclusions of this article will be made available by the authors, without undue reservation.

## Ethics Statement

The studies involving human participants were reviewed and approved by The Institutional Review Board of Ethics at Tel Aviv University. The patients/participants provided their written informed consent to participate in this study.

## Author Contributions

All authors contributed to this work to a significant extent, have read the article and agreed to submit it for publication after discussing the results and implications and commented on the article at all stages, and are, therefore, responsible for the reported research and have approved the final article as submitted.

## Conflict of Interest

The authors declare that the research was conducted in the absence of any commercial or financial relationships that could be construed as a potential conflict of interest.

## Publisher’s Note

All claims expressed in this article are solely those of the authors and do not necessarily represent those of their affiliated organizations, or those of the publisher, the editors and the reviewers. Any product that may be evaluated in this article, or claim that may be made by its manufacturer, is not guaranteed or endorsed by the publisher.

## Appendix

### Appendix A

Three-word sentences including a subject, a predicate, and an object (mean duration = 104.67 ± 6.11 msec) were used for the VD test. The sentences were shortened from the original 5-word sentences from the Hebrew version of the Matrix test to three words in order to minimize working memory demands. Sentences were manipulated using a 13-point stimulus continuum, exponentially ranging in √2 steps from a change of −0.18 semitone to a change of −8 semitones. This manipulation resulted in the following three separate dimensions: (1) F0, (2) formant frequencies, in which all formants were shifted down by an equal ratio, according to the 13-point stimulus continuum, and (3) combined, F0 + formants, in which both F0 and formants were shifted down in a similar manner. For example, the mean F0 for the F0-manipulated sentences varied by 0, −0.18, −0.26, −0.36, −0.51, −0.72, −1.02, −1.44, −2.02, −2.86, −4.02, −5.67, and − 8 semitones from the original sentence mean F0. Consequently, for the first sentence, the mean F0 was 175.62 Hz and the comparison sentences changed exponentially in √2 steps from 174 to 110.35 Hz, using the PSOLA algorithm (Moulines and Charpentier, 1990) for pitch extraction and manipulation. The formant frequencies for this sentence were manipulated exponentially, ranging in √2 steps from 0.99 (the smallest ratio between the original formant frequencies and the manipulated formant frequencies) to 0.63 (the highest ratio). This manipulation required resampling the stimulus to compress the frequency axis by a range of factors similar in ratio to the F0 change. The PSOLA algorithm was then applied to regain the original pitch and duration. Given that the average VTL of an adult female is approximately 140 mm (Fitch and Giedd, 1999), and formant frequencies are inversely proportional to VTL (Lammert and Narayanan, 2015), the formants range corresponded to a change from 2 to 88 mm. Note that this is a wider VTL range than expected from humans and was originally chosen to avoid floor effects for CI participants who had difficulty in perceiving normal difference limen (DL) for VTL (Zaltz et al., 2018). All manipulations were implemented using the PRAAT software version 5.4.17 (copyright 1992–2015 by Boersma and Weenink). Spectrographic displays for one of the sentences that was manipulated in F0, formants and F0 + formants for the VD test can be found in Zaltz et al. (2020).
